# Analysis of local extracellular matrix identifies different aetiologies behind bicuspid and tricuspid aortic valve degeneration and suggests therapies

**DOI:** 10.1007/s00018-023-04926-1

**Published:** 2023-08-26

**Authors:** Christian M. Beusch, Oscar E. Simonson, Johan O. Wedin, Pierre Sabatier, Ulrika Felldin, Sandeep Kadekar, Cecilia Österholm, Ákos Végvári, Roman A. Zubarev, Karin Fromell, Bo Nilson, Stefan James, Elisabeth Ståhle, Karl-Henrik Grinnemo, Sergey Rodin

**Affiliations:** 1 Division of Chemistry I, Department of Medical Biochemistry and Biophysics, Karolinska Institutet, 171 77 Stockholm, Sweden; 2 Cardio-Thoracic Translational Medicine (CTTM) Lab, Department of Surgical Sciences, Uppsala University, 752 37 Uppsala, Sweden; 3 Department of Cardio-Thoracic Surgery and Anesthesia, Uppsala University Hospital, 751 85 Uppsala, Sweden; 4 Novo Nordisk Foundation Center for Protein Research, University of Copenhagen, 2200 Copenhagen, Denmark; 5 Department of Molecular Medicine and Surgery, Karolinska Institutet, 171 77 Stockholm, Sweden; 6 Rudbeck Laboratory, Department of Immunology, Genetics and Pathology, Uppsala University, 751 85 Uppsala, Sweden; 7 Department of Medical Sciences, Uppsala University, 752 37 Uppsala, Sweden

**Keywords:** Aortic stenosis/regurgitation, Extracellular matrix, Proteomics

## Abstract

Aortic valve degeneration (AVD) is a life-threatening condition that has no medical treatment and lacks individual therapies. Although extensively studied with standard approaches, aetiologies behind AVD are unclear. We compared abundances of extracellular matrix (ECM) proteins from excised valve tissues of 88 patients with isolated AVD of normal tricuspid (TAV) and congenital bicuspid aortic valves (BAV), quantified more than 1400 proteins per ECM sample by mass spectrometry, and demonstrated that local ECM preserves molecular cues of the pathophysiological processes. The BAV ECM showed enrichment with fibrosis markers, namely Tenascin C, Osteoprotegerin, and Thrombospondin-2. The abnormal physical stress on BAV may cause a mechanical injury leading to a continuous Tenascin C-driven presence of myofibroblasts and persistent fibrosis. The TAV ECM exhibited enrichment with Annexin A3 (*p* = 1.1 × 10^–16^ and the fold change 6.5) and a significant deficit in proteins involved in high-density lipid metabolism. These results were validated by orthogonal methods. The difference in the ECM landscape suggests distinct aetiologies between AVD of BAV and TAV; warrants different treatments of the patients with BAV and TAV; elucidates the molecular basis of AVD; and implies possible new therapeutic approaches. Our publicly available database (human_avd_ecm.surgsci.uu.se) is a rich source for medical doctors and researchers who are interested in AVD or heart ECM in general. Systematic proteomic analysis of local ECM using the methods described here may facilitate future studies of various tissues and organs in development and disease.

**Supplementary Information:**

The online version contains supplementary material available at 10.1007/s00018-023-04926-1.

## Introduction

Aortic valve degeneration (AVD) represents the third most common cardiovascular disease in the Western world [1]. It currently has no medical treatment, leads to impaired function of the aortic valve, and warrants surgical or interventional aortic valve replacement (AVR). Aortic valve degeneration manifests in aortic stenosis, aortic regurgitation, or a mixture of the two. Degeneration of the tricuspid aortic valve (TAV) is the most common aetiology behind aortic stenosis [1] and mainly affects older patients. Bicuspid aortic valve (BAV) is the most common congenital heart disease. It is a result of a disturbed valvulogenesis [2], which leads to fusion of the aortic valve cusps, sometimes with a raphe between them. Depending on the pattern of the fusion and anatomical orientation of the commissures, BAV is classified into five types [3]. Although BAV is a feature of several genetic syndromes, the majority of cases are non-syndromic, have a complex polygenic origin and are inherited with incomplete penetrance and variable expressivity of the phenotype [4]. In comparison with the degeneration of TAV, BAV predisposes to aortic stenosis in younger patients [5]. Although the prevalence of BAV is about 1–2% in the general population [6], BAV patients represent half of all patients that undergo surgical AVR for aortic stenosis [5]. The degenerative processes, especially in combination with congenital BAV, are also the main cause behind the less prevalent aortic regurgitation [7].

The molecular cues that trigger degenerative processes in TAV and BAV are not fully understood. Degeneration of TAV is associated with the presence of cardiovascular risk factors, including coronary artery disease [8], while BAV has an autosomal dominant inheritance with incomplete penetrance and various expressivity [4]. Although significantly younger and under significantly fewer cardiovascular risk factors, the patients with BAV stenosis have a worse preoperative left ventricular function and a higher risk of post-operative heart failure than patients with TAV [9]. It suggests a possible difference in the molecular basis for the degeneration of TAV and BAV. Understanding the molecular basis might lead to development of medical treatments that postpone or replace AVR.

There is a large body of evidence that extracellular matrix (ECM) may be actively involved in the pathophysiology of localized diseases, for instance in the lungs [10], heart [11], and brain [12]. In contrast to a long-lasted earlier conception, ECM is not an inert scaffold but currently is emerging as a fixed in space, signalling, and dynamic entity, which is in constant crosstalk with the local cellular and immunological milieu [13, 14]. We hypothesize that ECM of the aortic valves, similar to fossils, accumulates molecular clues of the past pathophysiological events leading to AVD, and analysis of the ECM may reveal the aetiology behind the degeneration of BAV and TAV.

To test our hypothesis and further investigate the heterogeneity of AVD, we have initiated an observational experimental clinical study, which includes detailed anatomical classification of the aortic valves and proteomic characterization of the ECM from the valve tissues from patients undergoing open heart surgery for isolated AVD.

## Materials and methods

### Patients

In this study, we included 88 patients with severe AVD who were scheduled for surgical AVR at a tertiary-level referral centre (Uppsala University Hospital, Uppsala, Sweden). The diagnosis had been established prior to referral, and all patients had symptomatic severe aortic stenosis or aortic regurgitation as the primary indication for surgery. Patients with aortic regurgitation caused by active or previous endocarditis were excluded. Patients with a history of coronary artery disease, other valvular disease or previous heart surgery were not eligible for inclusion. All included patients received oral and written information about the study and provided written informed consent.

### ECM sample preparation from aortic valves and mass-spectrometry analysis

The aortic valve morphology was determined perioperatively by the surgeon and documented directly in the operating room. Bicuspid aortic valves were classified according to standard definitions [3]. Three patients had a rare unicuspid aortic valve morphology [5].

The aortic valve leaflets were cut into 5 × 5 mm pieces directly after excision. The leaflets were stored at − 80 °C in Allprotect Tissue Reagent (Qiagen, USA). ECM from frozen tissues was enriched using Compartment Protein Extraction Kit (Merck, Germany) according to the manufacturer’s instructions, including alkylation and deglycosylation [15]. Before digestion, the concentration of sodium dodecyl sulphate was increased to 3.5–4% to achieve complete solubilization of the ECM fractions. To digest the ECM samples, we used S-Trap™ technology (Protifi, USA) that has also been used by others for preparation of ECM samples [16] and other samples for mass spectrometry. Details of the sample preparation for mass spectrometry (MS) are available in the Extended Data. The samples for MS analyses were prepared blinded, and subsequent injection of samples for mass-spectrometry-based proteomics was performed in random order. Only after the raw data had been acquired, the study designer shared the key for data analysis. Details of the MS procedures and analyses are available in the Extended Data.

### Preparations of frozen sections of aortic valves

Frozen valve tissue samples stored in Allprotect Tissue Reagent (Qiagen, USA) were slowly thawed on ice and washed three times with ice-cold PBS, excessive liquid was removed by blotting the samples on Kimtech Science precision wipes (Merck, Germany) and the samples were cut in approximately 1-cm-long pieces. The pieces were placed inside Tissue-Tek Cryomold standard (Sakura, USA) cassettes, covered with OCT medium (Cryomount, Histolab, Sweden), and snap-frozen on dry ice. Every OCT block was cut into 5-μm-thick serial sections at around 50-μm intervals using CM1950 cryostat (Leica, Germany).

### Frozen sections of control (normal) valves

Frozen sections of the control (normal) valves were provided from Dr. Cecilia Österholm and had been prepared as described here [17]. In short, whole human hearts intended for biomedical research were obtained from the Legacy Donor Services Foundation, Florida, and processed at the Nova Southeastern University, Florida, USA. Written informed consent was obtained from the donor through registration as a tissue and organ donor with the Donate Life America, Donate Life Florida, or registering to be a donor when renewing or obtaining a driver’s license. Alternatively, written informed consent was obtained from the donor’s next-of-kin by a Donor Management Coordinator from the Legacy Donor Services Foundation. Inclusion criteria included donor’s age at death between 18 and 60 years old and no history of valvular heart disease. The hearts were processed within 48 h from the time of death. The aortic valves were inspected for absence of gross morphologic abnormalities; the cusps were excised at their origin and snap frozen in optimal cutting temperature compound. Control (normal) valve 1 belonged to 50-year-old male, and control (normal) valve 2 belonged to 55-year-old male.

### Immunostaining and immunohistochemistry (IHC)

Sections were fixed in 100% ice-cold MeOH, blocked with 5% goat serum and incubated overnight at 4 °C with primary antibodies of interest. For detection, MACH 2-conjugated polymer-alkaline phosphatase (AP) secondary antibodies (BioCare Medical, USA) were used together with Warp Red Chromogen Kit (BioCare Medical, USA). All the procedures were performed according to the manufacturers’ protocols. Sections were preserved in EcoMount mounting medium (BioCare Medical, USA) and examined under BX43 Olympus imaging system (Olympus, Tokyo, Japan). To visualize the histology of the valves, the frozen sections were stained with haematoxylin and 0.2% eosin (HE staining).

### Western blot

Alkylated and deglycosylated aortic valve ECM samples (procedure described above) were slowly thawed and kept on ice. SDS-PAGE Laemmli Loading buffer (BioRad, USA) with β-mercaptoethanol was added to the samples, and the mixtures were boiled at 100 °C for 5 min. The samples were resolved on SDS-PAGE 4–20% gradient gels and transferred to a polyvinylidene difluoride (PVDF) membrane. The membranes were blocked in 5% skimmed milk in Tris-buffered saline + 0.05% Tween20 (TBS-T; pH 7.6) and, after that, hybridized overnight with primary antibodies of interest. All washing steps were made with TBS-T except for the last step that was made with TBS. Proteins were visualized using Clarity Western ECL Substrate (BioRad, USA) and scanned using BioRad Chemi Doc Universal Hood II using the QuantiOne program.

### Quantification of western blots

Quantification of Western Blots was performed essentially as described by Aldridge et al. [18]. In short, for normalization for total protein input, additional SDS-PAGE 4–20% gradient gels were prepared as described above for Western Blots before the transfer, stained using SYPRO™ Ruby Protein Gel Stain (Thermo Scientific) according to the supplier’s instruction, and visualized using ChemiImager™5500 Fluorescence (Alpha Innotech, San Leandro, CA), and the images were acquired using ChemiImager5500 program Version 3.2.2. The densitometry analysis was performed using ImageJ 1.52a (National Institute of Health, USA). For relative quantification, the integrated density values were determined for equal-sized boxes (for each antibody) drawn around specific bands, with background values taken below in the same lanes from the same-sized boxes. The signals for each sample were calculated via subtraction of the background from the integrated density value around the specific band. In case the background value was higher than the integrated density value around the specific band, the signal was assumed to be equal to zero. For total protein stains, the integrated density values in equal-sized boxes around the entire lane were determined. The background values for the total protein quantification were taken in the same gels in the area without lanes to account for the unspecific staining of the gels.

### Antibodies

For immunostaining, antibodies against human Annexin A3 (Cat. number MA5-25327; Invitrogen/Thermo Fisher, USA), Tenascin C (Cat. number MA5-32128; Invitrogen/Thermo Fisher, USA), Apolipoprotein A-IV (Cat. number NBP1-86179; Novus Biologicals/Biotechne, USA), and myeloperoxidase (Cat. number HPA061464; Prestige Antibodies, Atlas Antibodies/Merck, USA) were taken at dilutions 1:200. For Western Blot analysis, the former two antibodies were taken at dilutions 1:500.

### Data availability

The mass-spectrometry proteomics data files have been deposited to ProteomeXchange Consortium (http://proteomecentral.proteomexchange.org) via the PRIDE partner repository [19] with the data identifier PXD025002. The analytic methods and study materials will be available to other researchers for purposes of reproducing the results or replicating the procedure upon request. Access to the aortic valve tissue and serum samples is limited due to the finite amount of the materials and restrictions imposed by the Ethical permits.

### Statistics

For baseline characteristics calculations, data were tested for normality using the Kolmogorov–Smirnov test with Lilliefors Significance Correction. Normally distributed continuous variables are expressed as mean values, while non-normally distributed values are expressed as median values. Categorical variables are presented as absolute numbers and percentages. Differences in continuous variables between BAV and TAV patients were assessed with Mann–Whitney *U* and independent-samples *t*-test as appropriate. Differences in categorical variables between BAV and TAV patients were analysed using the Chi-square test. The null-hypothesis was rejected when the *p*-value was < 0.05. All statistical analyses were performed with the use of SPSS software, version 27.0 (IBM).

For ECM mass-spectrometry data analysis, Proteome Discoverer (version 2.3, Thermo Scientific) using the SwissProt protein database with Mascot Server v 2.5.1 (MatrixScience Ltd., UK) search engine was used. The false discovery rate was set to 0.01 for peptide and protein level.

All data analyses and figures were made with R (version 4.0.2). Gene Ontology (GO) enrichment was performed with g:Profiler [20] using all annotated genes as background.

Protein intensities were normalized by variance stabilization normalization. The correlation between the patient samples was calculated using the Pearson correlation. All 88 aortic valves were used for tissue analysis.

Two-tailed Student’s *t* test (with equal or unequal variance depending on *F*-test) was applied to calculate *p*-values. No adjustments for multiple testing were made unless otherwise stated. The threshold for statistical significance was set for *p*-value < 0.01 and fold change (FC) > 1.5.

To determine the effect of AVD type and statin treatment on the protein levels, we used two-way analysis of variance (ANOVA) test followed by a Tukey’s honest significant difference test reporting adjusted *p*-values for multiple testing.

To analyse the absence and presence of individual proteins in certain AVD patient groups, we performed Fisher’s exact test and proteins were considered to be statistically significant if *p*-value < 0.01.

### Study approval

The study was approved by the local Ethics Review Board of Uppsala, Sweden (registration number 2017/221 and amendment 2017/221/1), and the study adhered to the Declaration of Helsinki.

## Results

### Reproducible ECM preparations

**Fig. 1:**
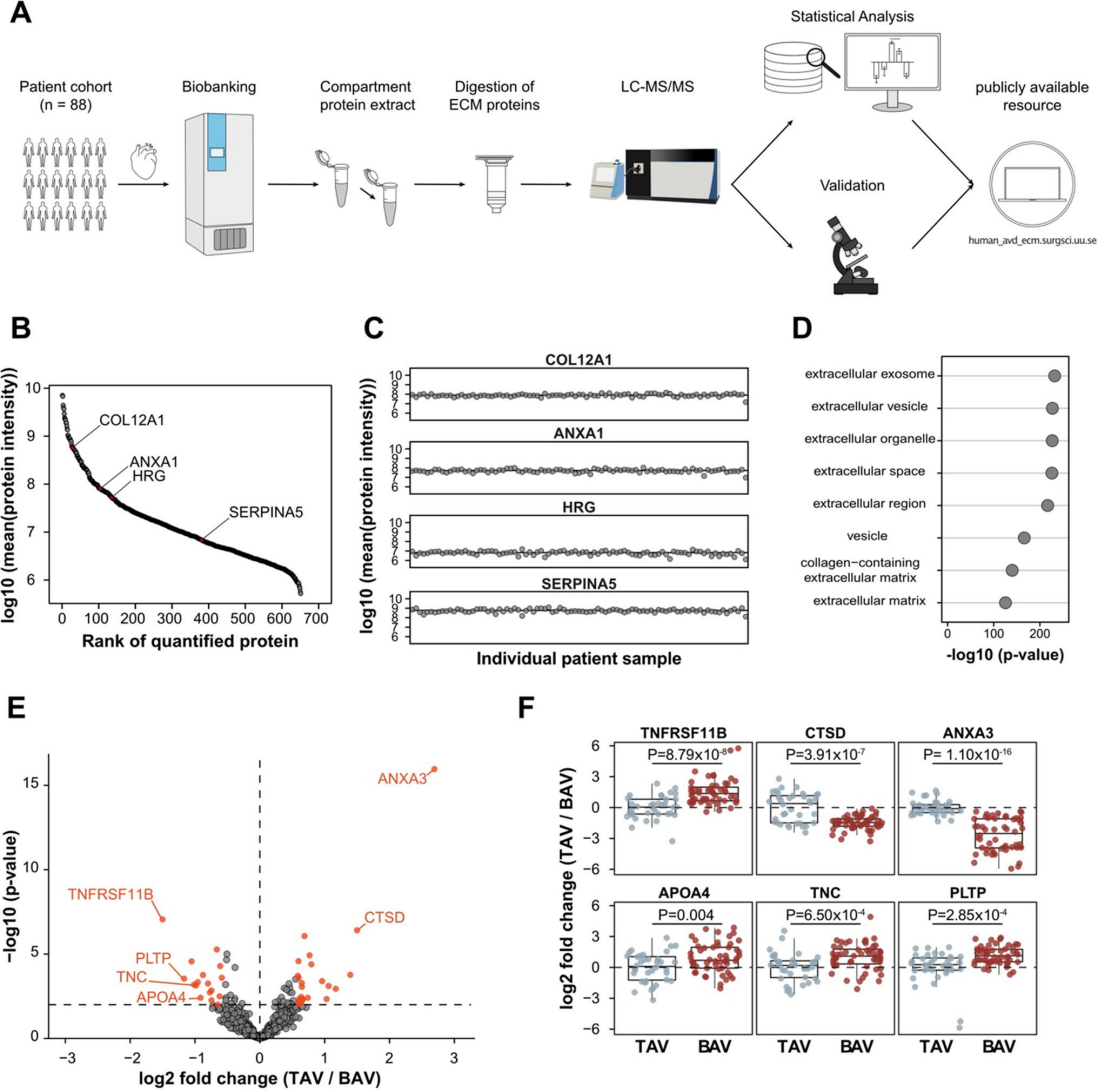
Workflow of the study, quality of the data and comparison of ECM in patients with TAV and BAV. **A** Workflow for the study. Aortic valve tissues from AVD patients with BAV and TAV were collected during open-heart surgery and stored in a biobank. The 88 samples were simultaneously processed using a compartment protein extraction kit. The ECM fractions were fully solubilized in 3.5–4% SDS, digested using S-Trap™ columns, protein abundances were quantified using label-free liquid chromatography tandem mass-spectrometry (LC–MS/MS), and the results were validated using independent methods. **B** Distribution of intensities for 651 quantified proteins (minimum 2 peptides per protein) with no missing values in the 88 samples. **C** Proteins with low CVs from different intensity ranges. **D** Enrichment analysis of the Gene Ontology (GO) cellular compartments among the quantified proteins. **E** Differential abundances of the 651 proteins in the TAV versus BAV group. Proteins that passed *p*-value *p* < 0.01 and fold change FC > 1.5 criteria are shown in red. **F** Several markers of BAV and TAV AVD ECM. The horizontal line in the box plots represents the median, 25th and 75th percentiles, and the whiskers represent measurements to the 5th and 95th percentiles. In figure, *GO* Gene Ontology; *AVD* aortic valve disease; *BAV* bicuspid aortic valve; *TAV* tricuspid aortic valve; *ECM* extracellular matrix; *CV* coefficient of variation; *TNFRSF11B* tumour necrosis factor receptor superfamily member 11B; *PLTP* phospholipid transfer protein; *TNC* Tenascin C; *APOA4* Apolipoprotein A-IV; *CTSD* Cathepsin D; *ANXA3* Annexin A3

Baseline characteristics of the whole cohort are summarized in Table 1*.* Among all 88 patients in the study (Fig. 1A), 36 had TAV, 33 had BAV type 1, 11 had BAV type 3, 5 had BAV of other types and three had unicuspid aortic valve (Supplementary Table 1). The median number of identified proteins was 1847 per ECM sample (ranging from 1478 to 2334), but only proteins with at least two peptides and no missing values were considered for downstream analysis resulting in 651 common proteins (Supplementary Table 2) that were detected in all the 88 samples spanning an abundance range of approximately four orders of magnitude (Fig. 1B). The median CV of all quantified proteins was 61% indicating high reproducibility of the sample preparation and analysis. All abundance ranges included proteins with CVs lower than the median value (Fig. 1C). Analysis of the Gene Ontology cellular compartments among the quantified proteins identified strong enrichment with ECM and ECM-related terms (Fig. 1D).

**Table 1:** Baseline characteristics

Characteristic	Bicuspid aortic valve (*n* = 52)	Tricuspid aortic valve (*n* = 36)	*p* value
*Clinical characteristics*			
Age (years)	64	71	0.001
Male gender [no. (%)]	40 (76.9)	21 (58.3)	0.063
Body-mass index (kg/m^2^)^a^	26.9	28.7	0.067
Systolic blood pressure (mmHg)	130	140	0.278
Diastolic blood pressure (mmHg)	80	70	0.076
Heart rate (beats per min)	69	69	0.828
Hypertension [no. (%)]	23 (44.2)	29 (80.6)	0.001
Diabetes mellitus [no. (%)]	5 (9.6)	8 (22.2)	0.101
Previous stroke [no. (%)]	6 (11.5)	0 (0.0)	0.035
Peripheral artery disease [no. (%)]	1 (1.9)	0 (0.0)	0.403
History of smoking [no. (%)]	21 (40.4)	17 (47.2)	0.524
Aortic aneurysm [no. (%)]	11 (21.2)	2 (5.5)	0.165
*Left ventricular ejection fraction*			
Good (≥ 51%) [no. (%)]	42 (80.7)	32 (88.9)	0.306
Moderate (31–50%) [no. (%)]	10 (19.2)	4 (11.1)	–
Poor (21–30%) [no. (%)]	0 (0.0)	0 (0.0)	–
Very poor (≤ 20%) [no. (%)]	0 (0.0)	0 (0.0)	–
*Medications*			
ACE inhibitor [no. (%)]	16 (30.8)	15 (41.7)	0.293
ARB [no. (%)]	8 (15.4)	13 (36.1)	0.025
Beta-blocker [no. (%)]	26 (50)	26 (72.2)	0.037
Calcium channel blocker [no. (%)]	10 (19.2)	14 (38.9)	0.042
Diuretics [no. (%)]	15 (28.8)	15 (41.7)	0.212
Statin [no. (%)]	12 (23.1)	20 (55.6)	0.002
*Laboratory characteristics*			
Haemoglobin (g/L)	143	142	0.843
Potassium (mmol/L)^a^	4.0	4.1	0.252
Glomerular filtration rate (mL/min/1.73 m^2^)^a^	77	68	0.002
NT-proBNP (ng/L)	424	554	0.828
Haemoglobin A1c (mmol/mol)	35	38	0.051

Data are expressed as median values and as numbers (percentage values)

*ACE* angiotensin-converting enzyme, *ARB* angiotensin II receptor blocker, *NT-proBNP* N-terminal pro-brain natriuretic peptide

^a^Normally distributed variables are presented as mean values

By comparing the abundances of the proteins in ECM samples from the patients with TAV with those from all other patients (BAV or unicuspid aortic valve) with a threshold for significance *p* < 0.01 and fold change, FC > 1.5, we found 40 proteins that passed the criterium (Table 2). In order to increase the accessibility of this unique proteomic data set, we provide an opportunity for a customized comprehensive analysis of the data in a dedicated online tool (human_avd_ecm.surgsci.uu.se). The publicly available tool allows to reproduce the results presented in this article but, additionally, to analyse the data stratified by type of the aortic valves including subtypes of BAV, by gender and by medications; to exclude patients from the analysis; and to visualize the correlation matrix and correlations of individual proteins of interest. The tool also allows easy export of the output data. The instructions for the web-based interface are presented in Supplementary Fig. 1.

**Table 2:** List of proteins that passed *p*-value *p* < 0.01 and fold change FC > 1.5 criterium in comparison of aortic valve ECM fractions of 36 TAV patients versus 52 BAV patients with aortic valve disease (no missing value allowed)

Uniprot accession	Symbol	Protein name	*p*-value	FC, log2(TAV/BAV)
P12429	ANXA3	Annexin A3	1.10 × 10^–16^	2.69
P07339	CTSD	Cathepsin D	3.91 × 10^–7^	1.50
Q00604	NDP	Norrin	1.71 × 10^–4^	1.39
P58166	INHBE	Inhibin beta E chain	0.0011	1.17
O14905	WNT9B	Protein Wnt-9b	7.76 × 10^–4^	1.06
Q02952	AKAP12	A-kinase anchor protein 12	0.0046	1.03
Q9H239	MMP28	Matrix metalloproteinase-28	4.04 × 10^–4^	0.95
P46939	UTRN	Utrophin	4.09 × 10^–5^	0.79
P39060	COL18A1	Collagen alpha-1(XVIII) chain	1.20 × 10^–5^	0.77
P27816	MAP4	Microtubule-associated protein 4	0.0039	0.74
P42224	STAT1	Signal transducer and activator of transcription 1-alpha/beta	8.55 × 10^–7^	0.69
Q8TAD2	IL17D	Interleukin-17D	0.0046	0.66
O43175	PHGDH	D-3-phosphoglycerate dehydrogenase	0.0052	0.66
P78527	PRKDC	DNA-dependent protein kinase catalytic subunit	8.74 × 10^–4^	0.65
P31150	GDI1	Rab GDP dissociation inhibitor alpha	5.24 × 10^–4^	0.65
O60504	SORBS3	Vinexin	0.0035	0.64
Q53RD9	FBLN7	Fibulin-7	0.0079	0.64
P15502	ELN	Elastin	0.0061	0.62
Q14152	EIF3A	Eukaryotic translation initiation factor 3 subunit A	0.0046	0.61
P00738	HP	Haptoglobin	0.0092	0.60
O43301	HSPA12A	Heat shock 70 kDa protein 12A	2.58 × 10^–4^	0.60
Q14980	NUMA1	Nuclear mitotic apparatus protein 1	2.64 × 10^–5^	0.60
Q13425	SNTB2	Beta-2-syntrophin	1.98 × 10^–4^	0.59
P53396	ACLY	ATP-citrate synthase	2.55 × 10^–4^	− 0.59
P10301	RRAS	Ras-related protein R-Ras	5.18 × 10^–5^	− 0.61
Q76M96	CCDC80	Coiled-coil domain-containing protein 80	0.0032	− 0.62
O14498	ISLR	Immunoglobulin superfamily containing leucine-rich repeat protein	0.0099	− 0.65
Q00341	HDLBP	Vigilin	5.39 × 10^–6^	− 0.66
P21926	CD9	CD9 antigen	0.0014	− 0.74
P16112	ACAN	Aggrecan core protein	0.0055	− 0.75
P06756	ITGAV	Integrin alpha-V	0.0019	− 0.77
Q9Y240	CLEC11A	C-type lectin domain family 11 member A	5.57 × 10^–4^	− 0.80
Q92743	HTRA1	Serine protease HTRA1	1.74 × 10^–4^	− 0.88
P06727	APOA4	Apolipoprotein A-IV	0.0039	− 0.92
Q9UKZ9	PCOLCE2	Procollagen C-endopeptidase enhancer 2	4.66 × 10^–4^	− 0.96
P35442	THBS2	Thrombospondin-2	7.31 × 10^–4^	− 0.99
P24821	TNC	Tenascin C	6.50 × 10^–4^	− 1.02
P02792	FTL	Ferritin light chain	2.72 × 10^–5^	− 1.05
P55058	PLTP	Phospholipid transfer protein	2.85 × 10^–4^	− 1.17
O00300	TNFRSF11B	Tumour necrosis factor receptor superfamily member 11B	8.79 × 10^–8^	− 1.50

*BAV* bicuspid aortic valve, *TAV* tricuspid aortic valve

### Enrichment of fibrosis markers in the ECM of BAV patients

**Fig. 2:**
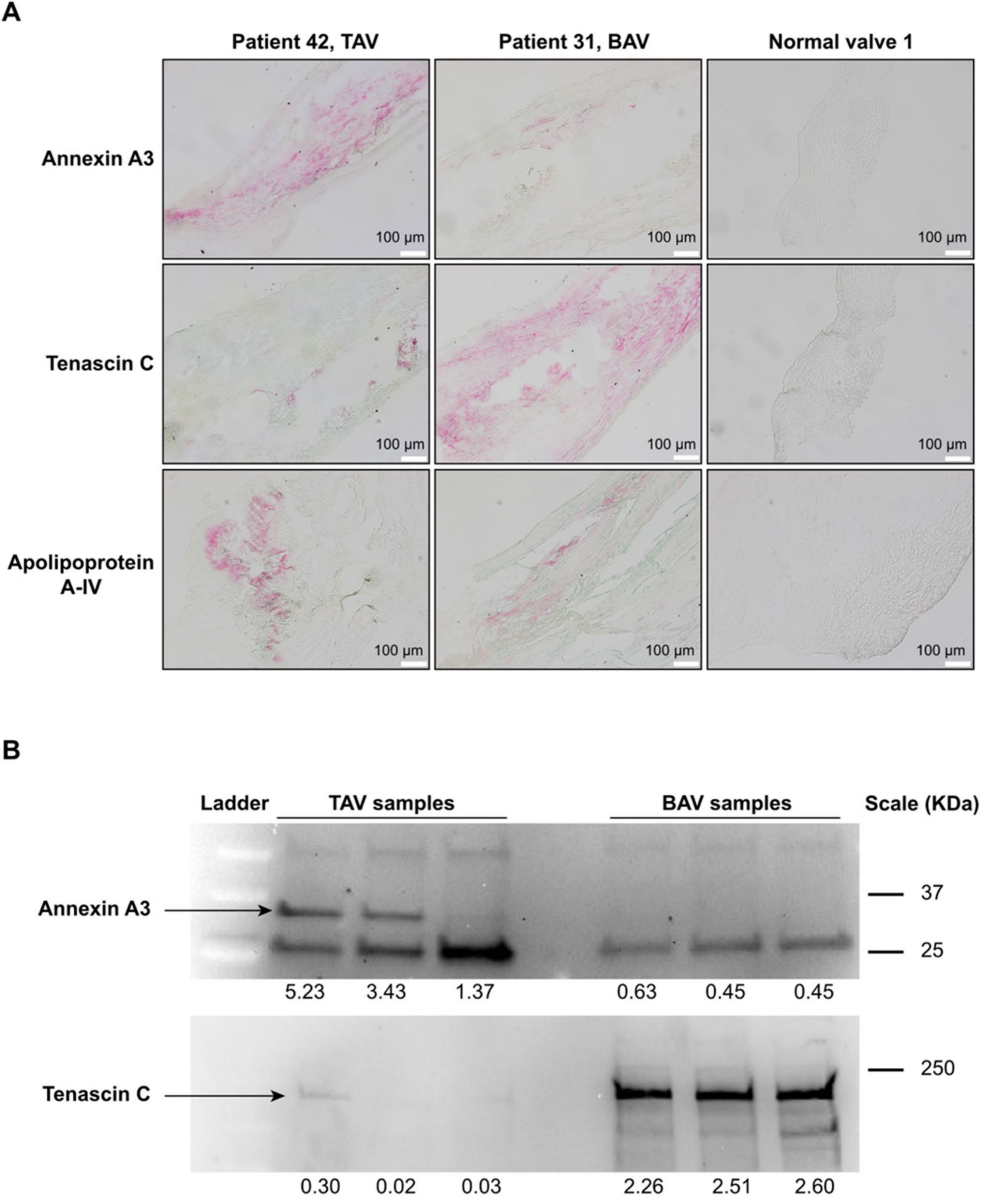
Confirmation of the mass-spectrometry data with Western blotting and immunostaining. **A** Immunostaining of aortic valve tissues of aortic valve degeneration patients (with TAV and BAV) and normal aortic valves (control) with anti-Annexin A3, anti-Tenascin C, and anti-Apolipoprotein A-IV. **B** Western blot analyses of Annexin A3 and Tenascin C abundances in ECM of AVD patients with TAV and BAV. Black arrows indicate specific bands for Annexin A3 and Tenascin C. Numbers below Western Blots represent relative quantification of the signals using total protein stain (Supplementary Fig. 9) for normalization of the sample input. In figure, *BAV* bicuspid aortic valve; *TAV* tricuspid aortic valve

**Fig. 3:**
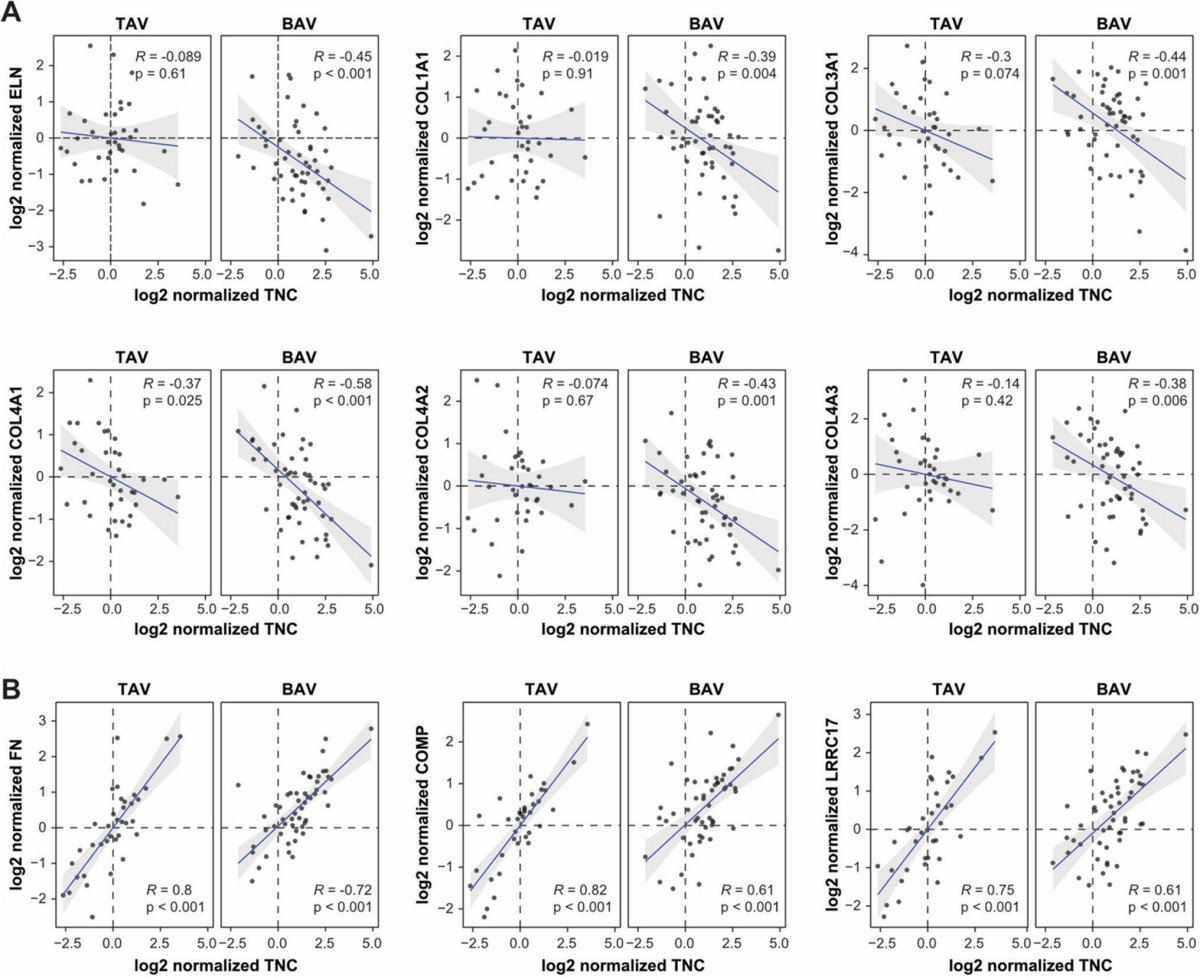
Correlation between abundance of Tenascin C and structural component of normal valves and fibrosis markers. **A** Correlation plots of structural ECM components normally present in healthy aortic valves. Elastin (ELN) and various collagen chains (COL1A1, COL3A1, COL4A1, COL4A2, and COL4A3) correlate negatively with Tenascin C (TNC) in the ECM of BAV patients, but to a lesser extent in the ECM of TAV patients. The letter *R* indicates the Pearson correlation coefficient, *p* indicates the Pearson *p*-value, and shaded regions denote 95% confidence intervals. **B** Correlation plots of fibrosis markers fibronectin (FN1) and cartilage oligomeric matrix protein (COMP) and a myofibroblasts marker Leucine-rich repeat-containing protein 17 (LRRC17) with the abundance of Tenascin C (TNC) in ECM of AVD patients that had the highest correlation in the both BAV and TAV groups. In figure, *BAV* bicuspid aortic valve; *TAV* tricuspid aortic valve; *ECM* extracellular matrix

In comparison with TAV patients, the ECM of BAV patients demonstrated a significant enrichment with the fibrosis marker Tenascin C (TNC) (Fig. 1E, F and Table 2), which has limited expression in healthy adult human tissues [21, 22]. The finding was validated by Western blot analysis of the ECM from 18 TAV and 18 BAV patients from the study cohort (Fig. 2B and Supplementary Fig. 4) and illustrated by immunostaining (Fig. 2A and Supplementary Fig. 3). Healthy aortic valves showed no staining with anti-TNC antibodies (Fig. 2B). The abundance of TNC negatively correlated with several structural proteins of the aortic valve in BAV patients, for instance elastin and some ubiquitous collagens (Fig. 3A). Only abundance of COL4A1 and COL4A3 exhibited a significant negative correlation (*p* < 0.05) with TNC in the TAV group (Fig. 3A). As expected, in both BAV and TAV groups, the abundance of TNC exhibited the strongest correlation with the fibrosis markers such as fibronectin and cartilage oligomeric matrix protein as well as a marker of myofibroblasts, Leucine-rich repeat-containing protein 17 [23] (Fig. 3B).

Also, the ECM of BAV patients was significantly enriched with fibrosis markers Thrombospondin-2 (THBS2) [24] and tumour necrosis factor receptor superfamily member 11B (TNFRSF11B, also called Osteoprotegerin) [25].

### Massive enrichment of Annexin A3 in the ECM of TAV patients

In TAV patients, Annexin A3 (ANXA3) exhibited the lowest statistical uncertainty (*p* = 1.1 × 10^–16^) and the highest fold change (FC = 6.5) among all the detected proteins (Fig. 1E, F and Table 2). The abundance of ANXA3 showed no correlation with age at surgery (Supplementary Fig. 2). Immunostaining of the frozen valve tissues with anti-ANXA3 antibodies was positive for AVD patients and negative for healthy controls (healthy aortic valves collected post-mortem from two people without aortic valve disease) (Fig. 2A and Supplementary Fig. 3). To confirm the mass-spectrometry data, we quantified the abundance of ANXA3 in the ECM from 18 TAV and 18 BAV patients from the study cohort using Western blot analysis, which confirmed the enrichment of ANXA3 in patients with TAV (Supplementary Fig. 4 and Fig. 2B). Neither immunostaining nor hematoxylin–eosin staining revealed any presence of neutrophils in the tissue of TAV (Supplementary Fig. 5), indicating no active inflammation.

### Different abundance of proteins involved in cholesterol deposition in the ECM of TAV and BAV patients

The ECM of BAV patients exhibited significantly higher abundances of several proteins involved in cholesterol metabolism: most importantly, phospholipid transfer protein (PLTP) [26], a high-density lipid (HDL)-metabolism regulator, and apolipoprotein A-IV (APOA4) (Fig. 2A and Supplementary Fig. 3), but also procollagen C-endopeptidase enhancer 2 (PCOLCE2) (Fig. 1E, F and Table 2), which is involved in HDL-cholesteryl ester uptake [27, 28] and the HDL-binding protein, Vigilin (HDLBP) [29]. Since statin treatment affects cholesterol levels in the blood and there was a higher incidence of statin treatment among the patients with TAV compared to that in the patients with BAV (Table 1), we performed a two-way ANOVA test and demonstrated that the difference in the ECM was not the result of the difference in the medication (Supplementary Fig. 6).

### Similar ECM landscape within BAV AVD cohort

**Fig. 4:**
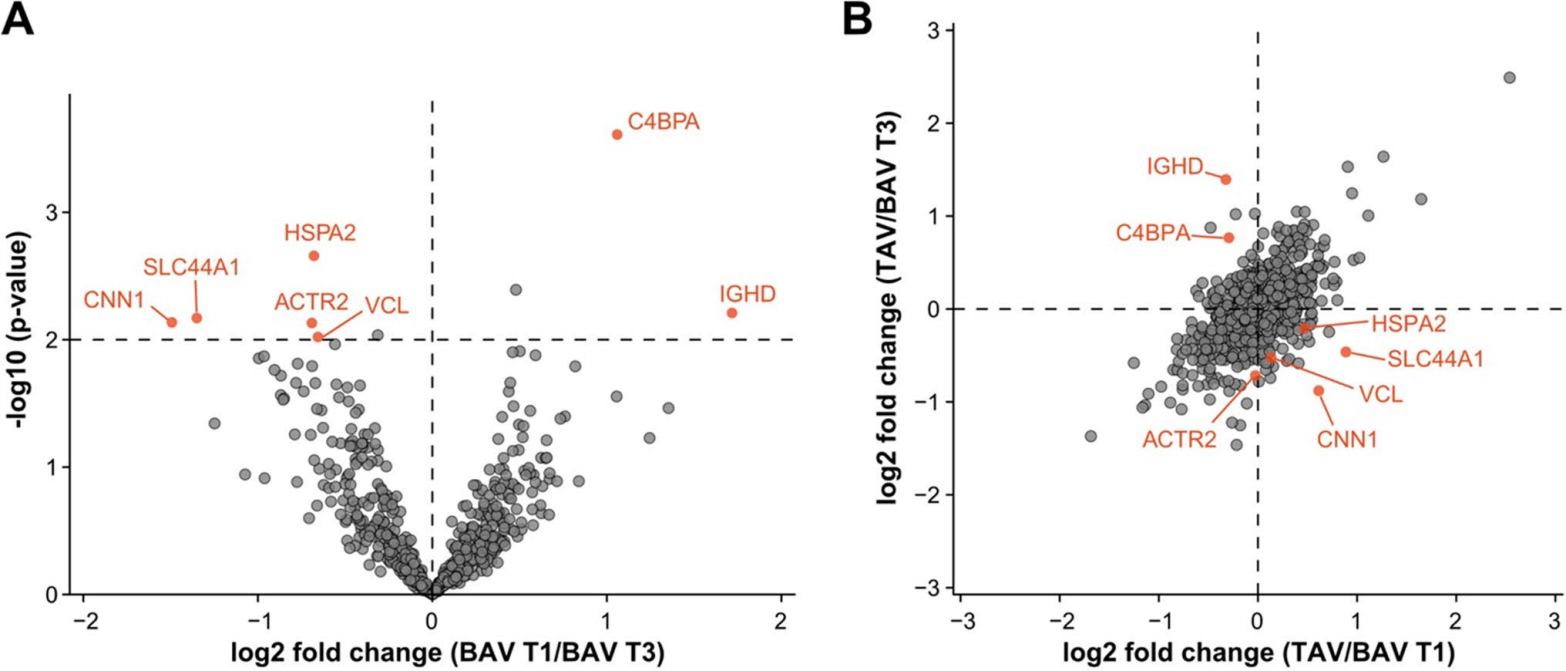
Comparison of ECM in patients with BAV type 1 and type 3. **A** Differential abundances of the 651 proteins in ECM of BAV type 1 versus BAV type 3 patients. Proteins that passed *p*-value *p* ≤ 0.01 and fold change FC ≥ 1.5 criterium are shown in red. **B** Correlation plot of relative ECM protein abundances of TAV versus BAV type 1 with TAV versus BAV type 3 group. In figure, *BAV* bicuspid aortic valve; *T1* type 1; *T3* type 3; *TAV* tricuspid aortic valve; *CNN1* Calponin 1; *SLC44A1* Solute Carrier Family 44 Member 1; *ACTR2* actin-related protein 2; *HSPA2* heat shock-related 70 kDa protein 2; *VCL* Vinculin; *IGHD* Immunoglobulin Heavy Constant Delta

To assess the heterogeneity of ECM inside the general BAV group (both stenosis and regurgitation), we compared the largest subgroups BAV type 1 (33 patients) and BAV type 3 (11 patients). The comparison exhibited only seven proteins with significantly different abundances (Fig. 4A and Supplementary Table 3) as opposed to the 40 proteins that distinguished the BAV and the TAV groups. Noteworthy, BAV type 1 and type 3 showed similar ECM landscapes (Fig. 4A and B) in spite of the very diverse genetics of BAV [4].

### Similar changes in ECM in TAV and BAV patients within aortic stenosis and regurgitation subgroups

**Fig. 5:**
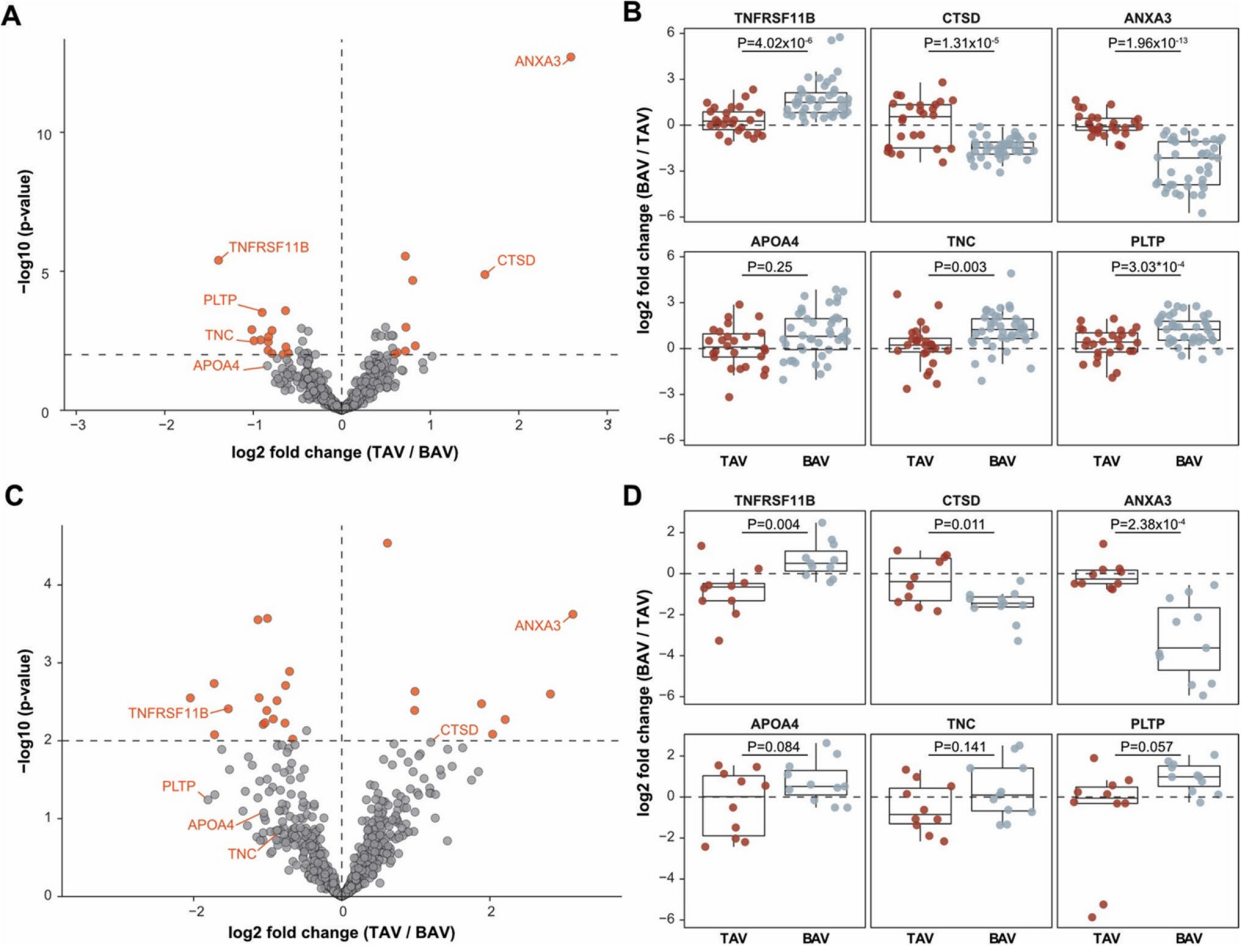
**A** Differential abundances of the 651 proteins in TAV versus BAV in the aortic stenosis subgroup. Proteins that passed *p*-value *p* < 0.01 and fold change FC > 1.5 criteria are shown in red. **B** The markers of BAV and TAV from the general cohort (Fig. 1F) in the aortic stenosis subgroup. Box plots represent the median, 25th and 75th percentiles, and the whiskers represent measurements to the 5th and 95th percentiles. **C** Differential abundances of the 651 proteins in TAV versus BAV in the aortic regurgitation subgroup. Proteins that passed *p*-value *p* < 0.01 and fold change FC > 1.5 criteria are shown in red. **D** The markers of BAV and TAV from general cohort (Fig. 1F) in the aortic regurgitation subgroup. Box plots represent the median, 25th and 75th percentiles, and whiskers represent measurements to the 5th and 95th percentiles. In the aortic regurgitation subgroup, markers PLTP, CTSD, APOA4, and TNC did not pass the criterium for significance, but exhibited similar trends as in the aortic stenosis subgroup and the general cohort. In figure, *BAV* bicuspid aortic valve; *TAV* tricuspid aortic valve; *TNFRSF11B* tumour necrosis factor receptor superfamily member 11B; *PLTP* phospholipid transfer protein; *TNC* Tenascin C; *APOA4* Apolipoprotein A-IV; *CTSD* Cathepsin D; *ANXA3* Annexin A3

To validate our results for the two clinical entities, we analysed the 67 patients with aortic stenosis and the 21 patients with aortic regurgitation separately. In patients with aortic stenosis, a comparison of ECM samples from TAV (26 patients) and BAV (41 patients) revealed similar differences to those found in the general AVD cohort and all the markers mentioned above had significant differences in the abundances with the exception of Apolipoprotein A-IV that demonstrated a similar trend of enrichment (Fig. 5A, B and Supplementary Table 4). In patients with aortic regurgitation (10 TAV and 11 BAV patients), the markers Annexin A3, tumour necrosis factor receptor superfamily member 11B, and Vigilin reached the significance, while the others demonstrated similar trends of enrichment (Fig. 5C, D and Supplementary Table 5). The lack of statistical significance might be due to fewer patients in the aortic regurgitation subgroup.

### Analysis of proteins encoded by genes associated with development of BAV in the ECM

Out of all proteins encoded by genes that have been shown to be associated with development of BAV or BAV-related syndromes in humans [4], we detected tissue metallopeptidase inhibitor 1 (TIMP1), tissue metallopeptidase inhibitor 3 (TIMP3), Fibrillin 1 (FBN1), and Actin Alpha 2 (ACTA2). In comparison with TAV patients, the ECM of BAV patients demonstrated an enrichment with TIMP1 with a low statistical uncertainty (*p* = 6 × 10^–4^), but with only a slight difference in fold change (FC = 1.4). Thus, TIMP1 along with the other three proteins did not pass the criterium for significance defined in this paper (Supplementary Fig. 7A). We also compared the abundances of the four proteins within the BAV AVD cohort between the largest subgroups BAV type 1 (33 patients) and BAV type 3 (11 patients). None of the proteins passed the criterium for significance (Supplementary Fig. 7B).

## Discussion

Our study demonstrates that there is a significantly different ECM landscape of degenerated aortic valves in patients with BAV and TAV. The aim of the study was to compare two pathological conditions with each other, and enrichment or deficit is a relative term since the abundances of the proteins in the ECM of normal aortic valves are not known.

In the BAV patients, the ECM is enriched with the inflammatory and fibrotic markers, namely Tenascin C [30], Thrombospondin-2 [24], and Osteoprotegerin (tumour necrosis factor receptor superfamily member 11B) [25]. Osteoprotegerin is a marker of chronic fibrosis in patients with aortic stenosis [31] and predicts the progression of heart failure [32]. This is in concert with our recent finding that aortic stenosis patients with BAV have a higher risk of post-operative heart failure than the patients with TAV [9]. A previous study has demonstrated upregulation of Thrombospondin-2 expression in fibrotic and stenotic aortic valves, particularly in myofibroblasts, at the mRNA level [33], supporting the validity of our analysis performed on the protein level in the ECM of the valves. Expression of Tenascin C is under strict spatiotemporal control during development, and it is only expressed to a limited extent in normal healthy adult tissue, including the aortic valve, but reappears during tissue remodelling and resolution of injuries [21, 22]. One reason for such a strict control is the signalling function of Tenascin C that allows to recruit [34] and induce [30, 35] myofibroblasts from non-activated stromal fibroblasts, for instance via interaction with TLR4 [22, 30]. To corroborate with that, we show a correlation between the abundances of Tenascin C and a marker of myofibroblasts, Leucine-rich repeat-containing protein 17 (Fig. 3B), in the ECM of the aortic valves of all the patients with AVD. Expression of Tenascin C by myofibroblasts can create a vicious cycle that prevents the resolution phase of the wound-healing process and damages the tissue via excessive fibrosis formation. Knock-out for Tenascin C in animal models demonstrates accelerated inflammation and fibrosis resolution in many organs, including the cardiovascular tissues [21], suggesting that large depositions of the protein are responsible for the persistence of fibrosis [30]. Earlier pathological investigation of aortic stenosis patients has also revealed more prominent fibrosis in degenerated BAV compared with TAV [36].

Velocities and gradients of blood across the aortic valve are significantly higher in healthy young BAV individuals than in age-matched TAV controls causing abnormal physical stress of the tissue [37]. The rheological properties might be the reason for the initial mechanical injury of BAV. Taken together, these data suggest that the pathophysiology of degeneration of BAV may be initiated by a mechanical injury, which is not resolved due to excessive accumulation of Tenascin C leading to the persistent presence of myofibroblasts and formation of fibrotic scarring as a result. Targeting Tenascin C-dependent activation of fibroblast, for instance the TLR4 signalling axis, might become a therapeutic approach for delaying the degeneration of BAV.

Unexpectedly, the ECM of patients with TAV and BAV showed a significant difference in the abundance of proteins involved in cholesterol deposition, whereas TAV patients exhibited a significant deficit in HDL-metabolism proteins. Earlier prospective clinical trials of lipid-lowering therapies have not distinguished BAV and TAV patients, except for one trial [38] that demonstrated no effect on aortic stenosis progression [39–41]. In the future, prospective randomized trials with the separation of BAV and TAV patients into independent experimental groups need to be performed to compare the effects of lipid-lowering therapies on the progression of aortic stenosis and regurgitation.

We also demonstrate that the ECM of the TAV patients was strongly enriched with Annexin A3 in comparison with that in the BAV patients. The biological function of Annexin A3 is largely unknown [42]. Other Annexins, but not Annexin A3, have been shown to be involved in the development of atherosclerosis [43]. Since Annexin A3 is mainly expressed in neutrophils [44], we speculate that its accumulation might be a trace of an immune response to prior bacterial contamination of the valve, but this needs additional experimental confirmation. The involvement of pathogens in the development of aortic valve disorders has been discussed before (reviewed in [45]), but, so far, only proven for infectious endocarditis [46].

Our database also contains information on the proteins or mutations in peptides that are only present in subsets of samples and may contain information on peptides of bacterial and viral origin in ECM of the AVD patients. For instance, lysyl oxidase homolog 2 enzyme has been detected in 17 BAV patients, but not in a single TAV patient (Supplementary Fig. 8). The enzyme contributes to the development of scar tissue in various pathological conditions [47]. Targeting of the enzyme with the inhibitory antibody simtuzumab has been proven effective in mouse fibrosis models [47] and has been tested in clinical trials for scleroderma, idiopathic pulmonary fibrosis and cancer [13], and is, therefore, a promising candidate for the prevention of BAV degeneration.

We also analysed the abundances of proteins in relation to the genes that are suggested to be involved in development of BAV itself. Out of all such genes mentioned by Bravo-Jaimes and Prakash [4], we detected four of which only TIMP1 exhibited significant but subtle upregulation in AVD patients with BAV in comparison with that in AVD patients with TAV. Interestingly, none of the patients from the study had Turner’s syndrome that confers strong predisposition to development of BAV and is related to homozygosity for *TIMP1* gene. It has been shown that hemizygosity for *TIMP1* confers predisposition to BAV [48], but it is unclear whether the difference detected by us is related to the level of expression of the gene or to the pathophysiology of AVD in BAV.

To our knowledge, this is the first comprehensive atlas of ECM proteomics of the valve tissue from patients with isolated clinically significant aortic stenosis and regurgitation that separates AVD patients with BAV and TAV into independent experimental cohorts. Earlier studies of ECM in aortic stenosis have either analysed a small number of proteins or included few patients or studied ECM of the aorta [36, 49, 50]. In contrast, we provide an unbiased data-driven approach to the analysis of the database and a new insight into AVD via analysis of the ECM of the valvular tissue. The approach is complementary to standard analyses of biomarkers in blood and provides information that cannot be obtained using the standard approach. Although sharing similar phenotypic features at the time of aortic valve replacement, degeneration of TAV and BAV represents two distinct aetiologies that imply different therapeutic approaches. Pharmaceutical agents designed for the treatment of fibrosis via inhibition of Tenascin C-dependent activation of fibroblasts and/or inhibition of pro-fibrotic enzymes may be efficient in inhibiting degeneration of BAV. Since the ECM of TAV patients is depleted with HDL-metabolism markers, lipid-lowering therapy may be beneficial for TAV patients if initiated early in the degenerative process. To facilitate the accessibility of this unique proteomic database, we created an online tool (human_avd_ecm.surgsci.uu.se) for medical doctors and scientists who are interested in aortic valve degeneration and cardiovascular diseases in general. The tool allows for customized in-depth interrogation of the database for comparison of the ECM content in the AVD patients with aortic stenosis and/or regurgitation with TAV, all five types of BAV, and UAV providing information on the differences between any types of the valve and/or the clinical entity. Our work demonstrates the usefulness of systematic proteomic analysis of the local ECM and can serve as a model for future studies of various tissues and organs in development and disease.

### Supplementary Information

Below is the link to the electronic supplementary material.

Supplementary file1 (DOCX 2004 KB)

Supplementary file2 (XLSX 144 KB)

## Data Availability

The mass spectrometry proteomics data files have been deposited to ProteomeXchange Consortium (http://proteomecentral.proteomexchange.org) via the PRIDE partner repository [19] with the data identifier PXD025002.
