# Supplementary material for: Analysis of local extracellular matrix identifies different aetiologies behind bicuspid and tricuspid aortic valve degeneration and suggests therapies
Source: Cell Mol Life Sci. 2023 Aug 26;80(9):268. doi: 10.1007/s00018-023-04926-1 (PMC10460373; doi:10.1007/s00018-023-04926-1)
Supplement: Supplementary file 1 — Supplementary file1 (DOCX 2004 KB) [file 18_2023_4926_MOESM1_ESM.docx]

*Cellular and Molecular Life Sciences*

**Analysis of local extracellular matrix identifies different aetiologies behind bicuspid and tricuspid aortic valve degeneration and suggests therapies**

Christian M. Beusch*, Oscar E. Simonson*, Johan O. Wedin, Pierre Sabatier, Ulrika Felldin, Sandeep Kadekar, Cecilia Österholm, Ákos Végvári, Roman A. Zubarev, Karin Fromell, Bo Nilson, Stefan James, Elisabeth Ståhle, Karl-Henrik Grinnemo*, Sergey Rodin*†

*Contributed equally

†**To whom correspondence should be addressed:**

Sergey Rodin, associate professor
Department of Surgical Sciences
Uppsala University
752 37 Uppsala, Sweden
E-mail address: sergey.rodin@surgsci.uu.se

**SUPPLEMENTARY MATERIAL METHODS AND FIGURES**

**SUPPLEMENTARY METHODS**

**Enrichment of ECM Fractions in the Aortic Valve Samples**

Frozen valve tissue samples stored in Allprotect Tissue Reagent (Qiagen, US) were slowly thawed on ice, washed three times with ice cold PBS. Excessive liquid was removed by blotting the samples on Kimtech Science precision wipes (Merck, Germany) after which, the samples were cut into small pieces and weighed. Approximately 20-50 mg of a sample was used for the enrichment of the ECM fraction, following the manufacturers’ instructions of the Compartment Protein Extraction Kit (Merck, Germany)[1], which includes the deglycosylation step. The enrichment for one patient (patient number 79) was performed twice (samples 79 and 79re) to additionally test the reproducibility of the method (Supplementary Figure 10).

**Proteolytic Digestion on S-Trap Plate**

The ECM fraction samples were vortexed vigorously for 4-5 s and pipetted quickly up and down several times before shaking on a Vortex Genie disruptor at maximum speed (2850 rpm) for 5 min. An aliquot of 250 µL of each sample, corresponding to ca. 1 mm protein pellet as defined in manufacturer’s instruction to Compartment Protein Extraction Kit (Merck, Germany), was transferred to a 15 mL tube and supplemented with 50 µL solubilization buffer (20% SDS in 50 mM triethylammonium bicarbonate (TEAB), pH 7.55), 3 µL of cc. phosphoric acid and 2121 µL of binding buffer (100 mM TEAB in 90% methanol, pH 7.1) according to the manufacturer’s instructions (Protifi, Hungtington NY) with brief vortexing between each step. The whole sample volumes were loaded in 400 µL aliquots onto an S-Trap plate (Protifi, Hungtington NY), spun down at 1500 *g* for 2 min stepwise, followed by washing with 200 µL of binding buffer three times.

Proteolytic digestion was performed by adding 125 µL of 0.1 µg/µL trypsin (sequencing grade, Promega) in 50 mM TEAB incubated at 47°C for 1.5 h. The derived peptides were eluted consecutively applying 80 µL of 50 mM TEAB; 80 µL of 0.2% formic and 80 µL of 0.2% formic acid in 50% AcN with centrifugation at 1500 g for 2 min between each step and finally a drying step in a vacuum concentrator (miVac, Thermo Scientific). The samples were cleaned on a C18 Hypersep plate (Thermo Scientific) and dried in a 96-well plate to completeness and stored at -80°C.

**Mass-Spectrometry Analysis of ECM from Aortic Valves**

Prior to mass-spectrometry analysis, the samples were resuspended in 2% ACN and 0.1% FA (solvent A) and injected into an UltiMate 3000 UPLC autosampler (Thermo Scientific) coupled to an Orbitrap Fusion Tribrid mass-spectrometer (Thermo Scientific). The peptides were loaded on a trap column (Acclaim PepMap 100 C18, 100 μm × 2 cm) and separated on a 50 cm long C18 Easy spray column (Thermo Scientific). Chromatographic separation was achieved using the following gradient: 4-26% of solvent B (98% ACN and 0.1% FA) in 90 min, 26-95% in 5 min, and 5 min of 95%, before equilibration for 9 min at 4% with a flow rate of 300 nlmin^-1^. For data collection, the mass-spectrometer operated in positive polarity using a data-dependent acquisition (DDA) mode. The cycle time was 2 s and consisted of one full scan with a resolution of 120,000 (at 200 Th) covering the range from 350 to 1800 Th. Automatic gain control (AGC) was set to 4*10^5^ with a maximum injection time of 50 ms, and lock mass was set to 445.12003 Th. Triggered MS/MS scans were recorded with a resolution of 30`000, AGC of 50`000, maximum injection time of 54 ms, isolation window of 1.6 Th, and normalized collision energy (NCE) 30%. Only peptide with a charge from 2+ to 7+ were selected, dynamic exclusion was set to 45 s. All spectra were acquired in profile mode using the orbitrap.

Patient samples were randomized for the preparation and data collection for all LC-MS/MS analyses.

**Bioinformatics and Data Analysis of ECM from Aortic Valves**

MS raw files of DDA samples were analysed with Proteome Discoverer (version 2.3, ThermoFisher Scientific) and analysed using the SwissProt protein database with Mascot Server v2.5.1 (MatrixScience Ltd., UK) search engine. Parameters were chosen as follows: up to two missed cleavage sites for trypsin, peptide mass tolerance 10 ppm, 0.05 Da for the HCD fragment ions. Carbamidomethylation of cysteine was specified as a fixed modification, whereas oxidation of methionine, deamidation of asparagine and glutamine were defined as variable modifications. For quantification, both unique and razor peptides were allowed. The false discovery rate was set to 0.01 for peptide and protein level.

Only proteins with at least two identified peptides were included in the subsequent statistical analyses. All data analyses and figures were made with R (version 4.0.2). GO enrichment was performed with g:Profiler[2] using all annotated genes as background and all reliably quantified proteins as query input.

For further analysis, protein intensities were normalized by variance stabilization normalization (VSN). Coefficient of variation (CV) values of protein intensities were calculated according to the formula (sample standard deviation/ sample mean) and correlations analysis between the patients´ samples were calculated using Pearson correlation.

For statistical analyses, the extracted second time sample (patient number 79re) was removed from the analysis, protein intensities were log_2_ transformed and normalized by the average of all TAV samples. Two-tailed Student’s *t*-test (with equal or unequal variance depending on F-test) was applied to calculate p-values. Proteins were considered to be statically significant if p-value <0.01 and fold-change > log_2_(1.5). All 88 unique patient samples were used for data analysis.

Literature:

1. Naba A, Clauser KR, Hynes RO (2015) Enrichment of extracellular matrix proteins from tissues and digestion into peptides for mass spectrometry analysis. J Vis Exp 2015:e53057. https://doi.org/10.3791/53057

2. Reimand J, Kull M, Peterson H, et al (2007) G:Profiler-a web-based toolset for functional profiling of gene lists from large-scale experiments. Nucleic Acids Res 35:W193. https://doi.org/10.1093/nar/gkm226

**SUPPLEMENTARY FIGURES**


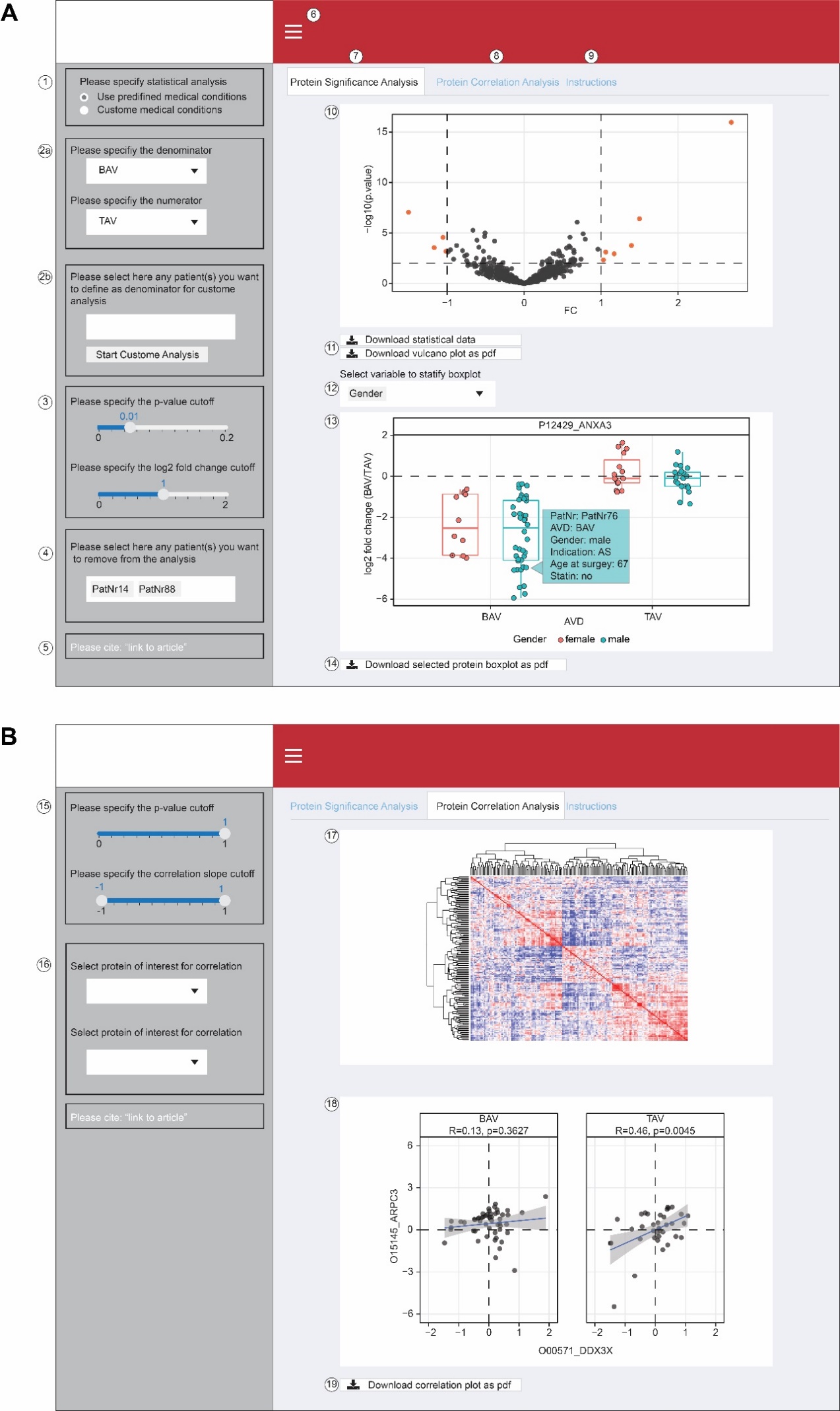


**Supplementary Figure 1. Instructions for the web interface.**

**A**, Step by Step instructions for the Protein Significance Analysis tab.

1) The user can decide to perform the statistical analysis based on predefined medical conditions or on custom grouping.

2a) If predefined medical conditions are selected in panel 1, the user can choose based on which patient medical record data the statistical analysis should be performed.

2b) If custom analysis is selected, the user can select patients, which should be used as denominator. To facilitate the analysis, the user can start with the predefined medical conditions if desired. Afterwards, the user is required to press the start custom analysis button.

3) The user can specify the p-value and log2 fold change thresholds for proteins to be highlighted in the volcano plot.

4) If needed, the user can exclude individual patients from the statistical analysis.

5) Link to the original article.

6) Hide or show the checkbox column.

7) Tab for the Protein Significance Analysis based on patient medical records.

8) Tab for the Protein Correlation Analysis of AVDs.

9) Tab which contains the instructions and guidance for the web interface.

10) An interactive volcano plot is created based on the user's input of the statistical analysis. By clicking on a protein, a boxplot with jitter with the individual patient data is created (see panel 12).

11) The user can download the statistical analysis as a tsv file and export the volcano plot as a pdf file.

12) If selected, the user can stratify the boxplot below by various patients’ characteristics.

13) An interactive boxplot with jitter is displayed if the user clicks on a protein in the volcano plot. Each dot represents an individual sample and, when hovered over, it displays the patients’ medical data.

14) The user can export the protein boxplot as a pdf file.

**B**, Step by Step instructions for the Protein Correlation Analysis tab.

15) The user can apply a cutoff for the p-value and the correlation slope, based on which the correlation heatmap (panel 16) is rendered.

16) The user can search and select specific protein pairs to be visualized in panel 17.

17) An interactive correlation heatmap is created, in which the bottom triangle represents the patients with BAV and the upper the patients with TAV. Hovering over a tile shows the corresponding proteins and the Pearson correlation.

18) By clicking on a specific tile in the heatmap or selecting two proteins in panel 15, the user can visualize the Pearson correlation between two proteins for BAV and TAV AVD.

19) The user can export the protein correlation plot as a pdf file.

In figure, AVD: aortic valve degeneration; BAV: bicuspid aortic valve; TAV: tricuspid aortic valve.


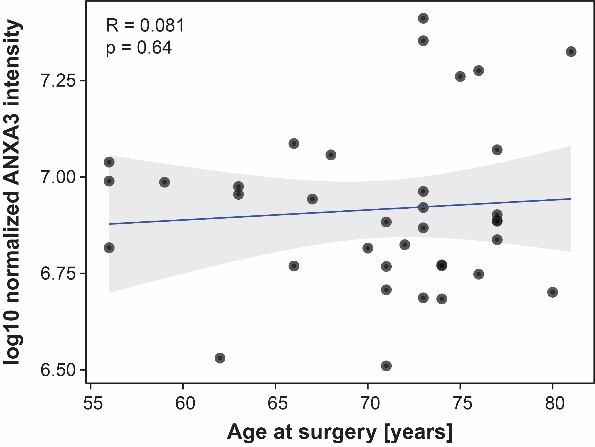


**Supplementary Figure 2.**

The absence of significant Pearson correlation between ANXA3 expression and age at surgery in ECM of aortic valve degeneration patients with tricuspid aortic valves. The letter R indicates the Pearson correlation coefficient, p indicates the Pearson p-value, and shaded regions denote 95% confidence intervals.


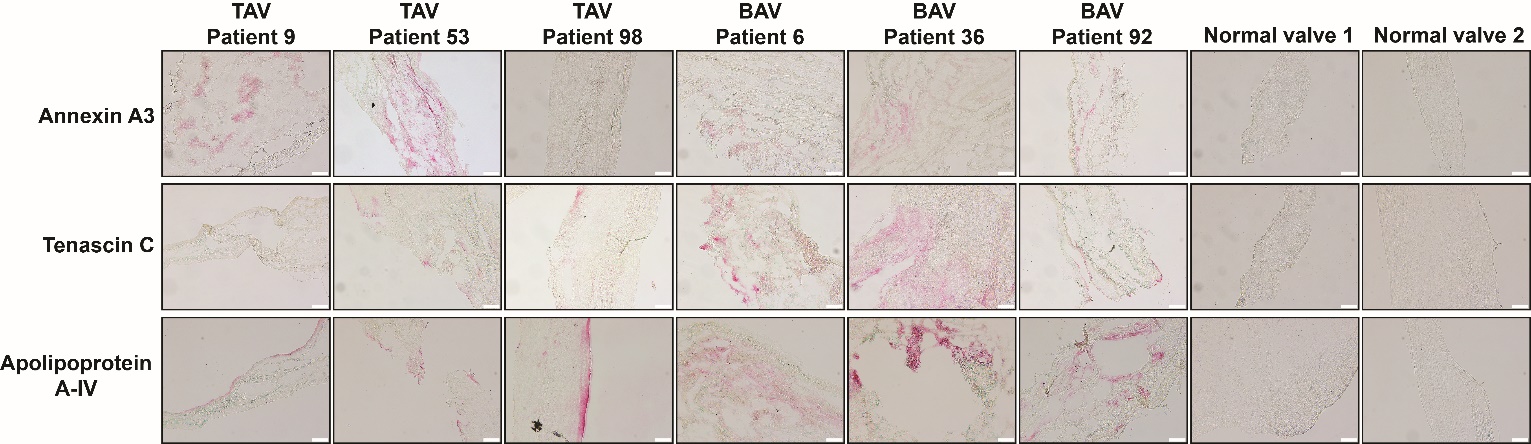


**Supplementary Figure 3.**

Representative immunostaining of aortic valve tissues of aortic valve degeneration patients with tricuspid aortic valve (TAV) and bicuspid aortic valve (BAV) and normal aortic valves (control) with anti-Annexin A3, anti-Tenascin C, and anti-Apolipoprotein A-IV. Scale bars – 100 μm.


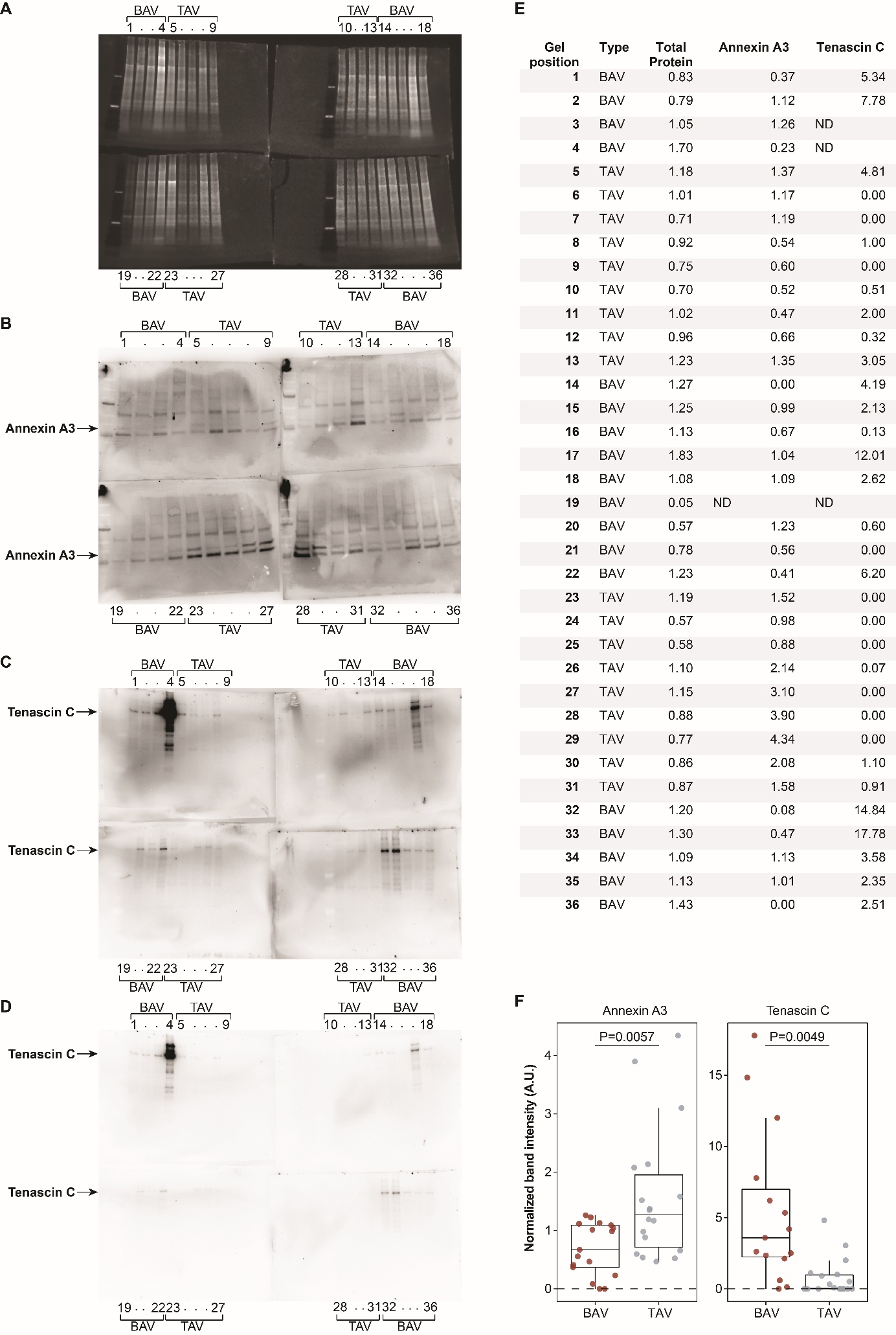


**Supplementary Figure 4.** **Quantification of relative abundances in the ECM samples of 18 TAV and 18 BAV patients with aortic valve degeneration.**

**A**, SYPRO Ruby total protein stain of the samples that was used for the quantification. **B**, Western blot analyses of Annexin A3 in ECM of AVD patients with TAV and BAV that was used for the quantification. Black arrows indicate the specific band for Annexin A3. **C**, Western blot analyses of Tenascin C abundances in ECM of AVD patients with TAV and BAV that was used for the quantification. Black arrows indicate the specific band for Tenascin C. **D**, Same as C but with lower exposition time. **E**, Relative quantification of total proteins, Annexin A3 and Tenascin C in the 18 TAV and 18 BAV patients. The total protein quantification is normalized to the mean signal in all the 36 samples. The BAV sample in lane 19 shown an aberrant low value of 0.05 and was excluded from the downstream analysis as an outlier. The Annexin A3 and Tenascin C signals were normalized to the respective median and total protein stain for the sample input. The quantification of Tenascin C signals for BAV samples in lanes 3 and 4 could not be performed because of the saturation of the signal. But, as shown in panel D the abundance of Tenascin C in the samples was higher than that in samples of patients with TAV. **F**, Graphic representation of the results from panel E. Horizonal line in the box plots represent the median, 25th and 75th percentiles and whiskers represent measurements to the 5th and 95th percentiles. In figure, ND: not defined; BAV: bicuspid aortic valve; TAV: tricuspid aortic valve.


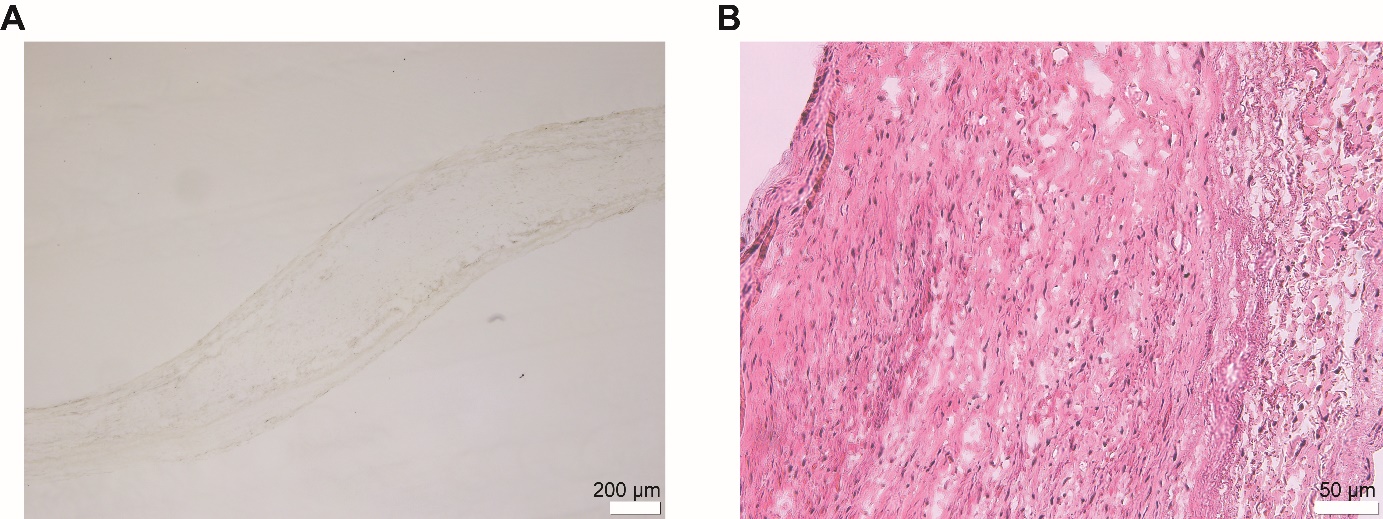


**Supplementary Figure 5.**

**A**, Representative immunostaining of aortic valve tissues of TAV AVD patients with anti-myeloperoxidase (MPO) antibodies (patient number 42 is shown). No positive signal was detected. **B**, Representative HE staining of aortic valve tissues of TAV AVD patients (patient number 42 is shown). No segmented nuclei that are characteristic for neutrophils were detected. AVD: aortic valve degeneration; TAV: tricuspid aortic valve.


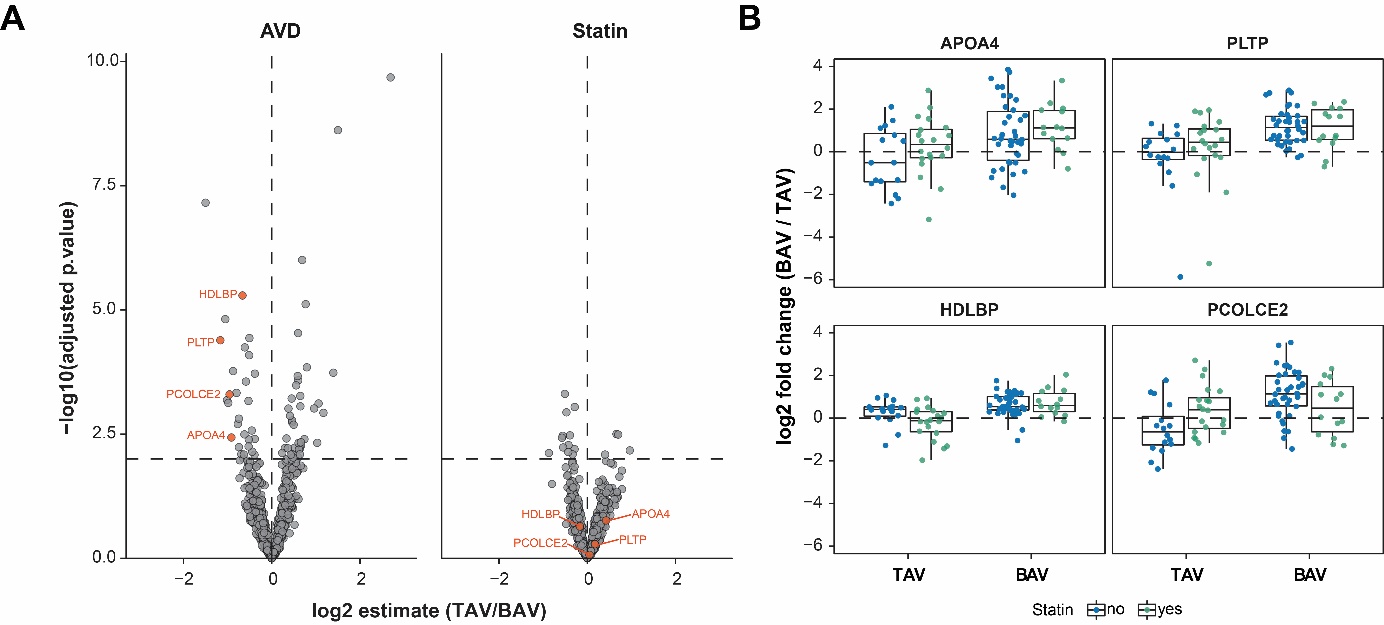


**Supplementary Figure 6. Differences in several cholesterol deposition markers are independent from statin intake.**

**A**, Two-way analysis of variance (ANOVA) with Tukey’s post-hoc test shows increased abundance of cholesterol deposition markers in the ECM of the patients with BAV comparted to that in the patients with TAV. **B**, Boxplot of several markers of cholesterol deposition grouped by TAV and BAV AVD and coloured based on the statin intake. Box plots represent the median, 25th and 75th percentiles and whiskers represent measurements to the 5th and 95th percentiles. In figure, AVD: aortic valve degeneration; BAV: bicuspid aortic valve; TAV: tricuspid aortic valve; APOA4: Apolipoprotein; PLTP: Phospholipid transfer protein; HDLBP: Vigilin; PCOLCE2: Procollagen C-endopeptidase enhancer 2.


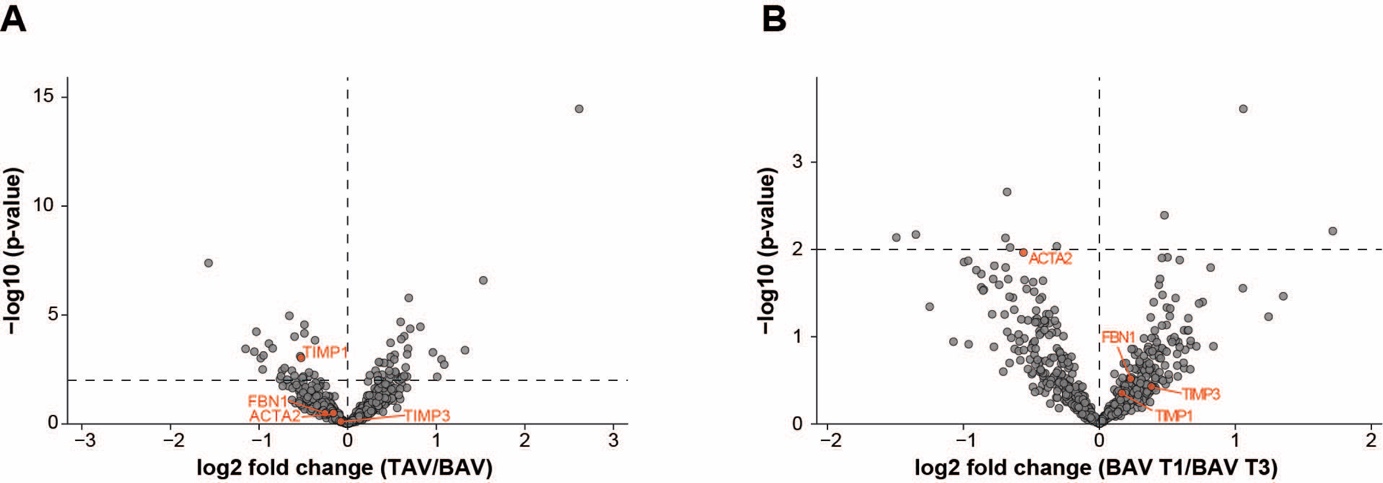


**Supplementary Figure 7. Abundance of Tissue metallopeptidase inhibitor 1 (TIMP1), Tissue metallopeptidase inhibitor 3 (TIMP3), Fibrillin 1 (FBN1), and Actin Alpha 2 (ACTA2) in the aortic valve ECM of the patients with AVD (shown in orange).**

**A**, Differential abundances of the four proteins in the TAV versus BAV group. TIMP1 had p=6*10^-4^ and FC=1.4. **B**, Differential abundances of the four proteins in the ECM of BAV type 1 versus BAV type 3 patients.


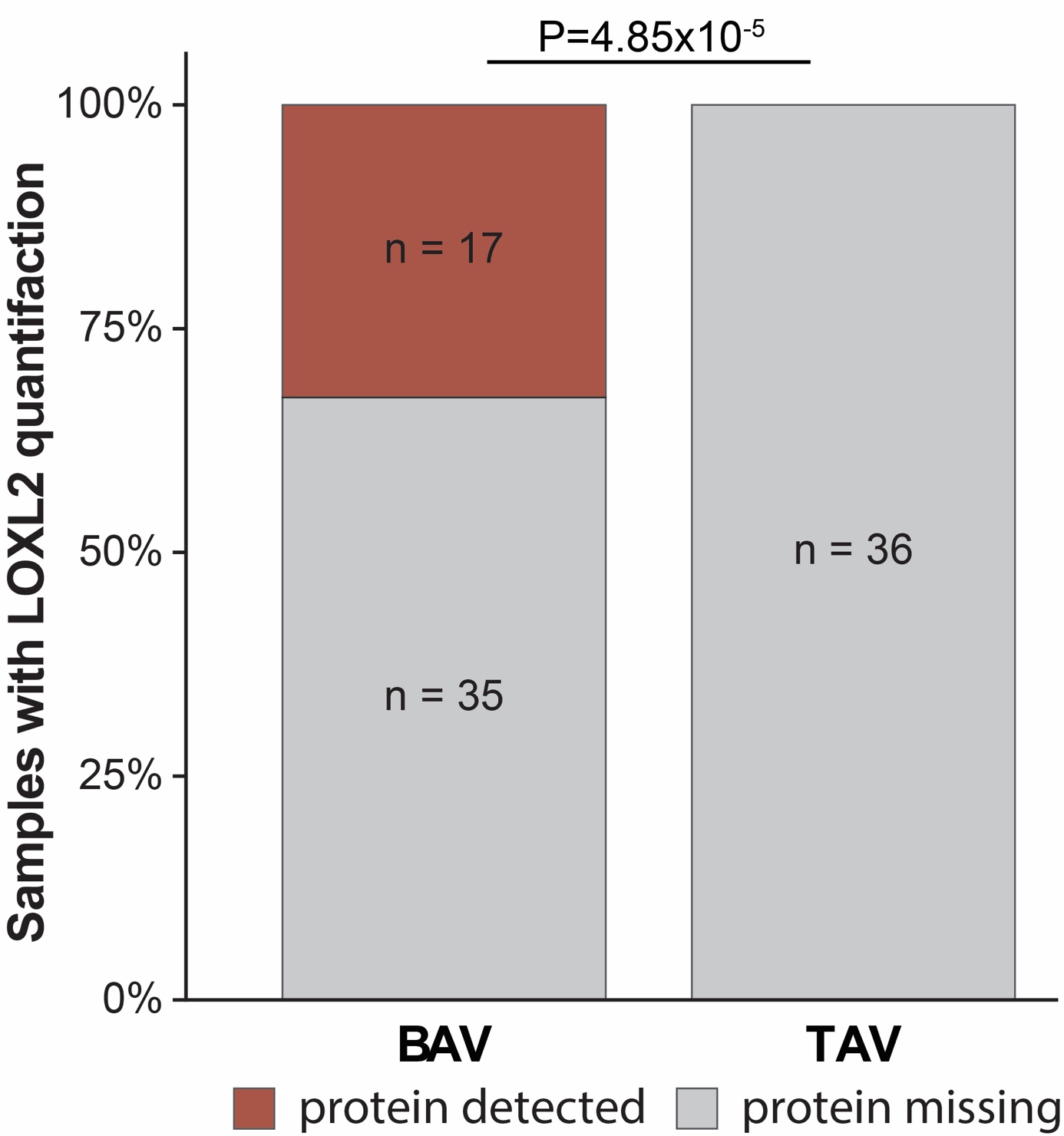


**Supplementary Figure 8. Absence and presence analysis of Lysyl oxidase homolog 2 enzyme (LOXL2) in the AVD patients with BAV and TAV.**

In figure, AVD: aortic valve degeneration; BAV: bicuspid aortic valve; TAV: tricuspid aortic valve; LOXL2: Lysyl oxidase homolog 2 enzyme.


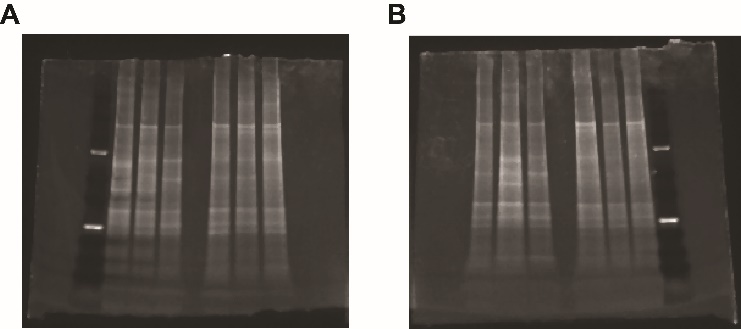


**Supplementary Figure 9.**

**A**, SYPRO Ruby total protein stain of the Annexin A3 Western Blot in Figure 2B. **B**, SYPRO Ruby total protein stain of the Tenascin C Western Blot in Figure 2B.


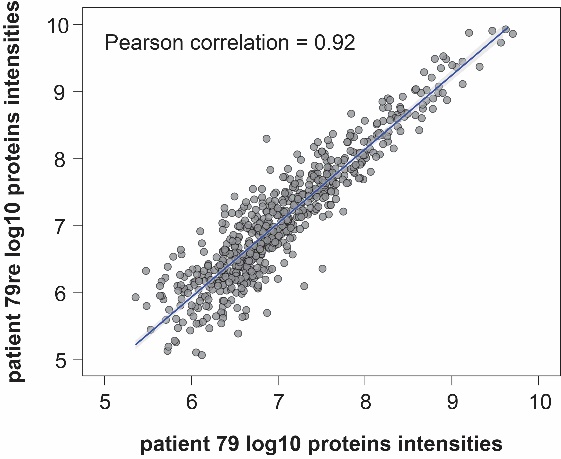


**Supplementary Figure 10.**

Correlation between the two extractions from aortic valve tissues of patient number 79 (79 and 79re) demonstrating high reproducibility of the ECM extraction protocol and LC-MS/MS analysis.
